# Supplementary material for: Conformational dynamics of the bacterial E3 ligase SspH1
Source: J Biol Chem. 2025 Sep 1;301(10):110671. doi: 10.1016/j.jbc.2025.110671 (PMC12506513; doi:10.1016/j.jbc.2025.110671)
Supplement: Supplementary Material [file mmc1.pdf]

Conformational dynamics of the bacterial E3 ligase SspH1

Cassandra R. Kennedy[1], Diego Esposito[1], Jessica Huber[1], David House[2] and Katrin Rittinger[1*]

[1]Molecular Structure of Cell Signalling Laboratory, The Francis Crick Institute, 1 Midland Road, London, NW1 1AT, United Kingdom.
[2]Crick-GSK Biomedical LinkLabs, GSK, Gunnels Wood Road, Stevenage, Hertfordshire, SG1 2NY, United Kingdom.

* Correspondence: katrin.rittinger@crick.ac.uk.

Contents

Supporting Figures S1-8
Table S1: X-ray data table
Table S2: SAXS data table

A

```
IpaH1.4  ---------------------------------------------------------------------
IpaH9.8  ---------------------------------------------------------------------
SspH1    -MFNIRN--TQPSVSMQAIAGAAAPEASPEEIVWEKIQVFFPQENYEEAQQCLAELCHPARGMLPDHIS  66
SspH2    MPFHIGSGCLPATISNRRIYRIAWSDTPPEMSSWEKMKEFFCSTHQTEALECIWTICHPPAGTTREDVI  69

IpaH1.4  -------------------------------------------------------------MIKSTNIQ   8
IpaH9.8  ---------------------------------------------------------------------
SspH1    SQFARLKALTFPAWEENIQCNRDGINQFCILDAGSKEILSITLDDAGNYTVNCQGYSEAHDFIMDTEPG 135
SspH2    NRFELLRTLAYAGWEESIHSGQHGENYFCILDEDSQEILSVTLDDAGNYTVNCQGYSETHRLTLDTAQG 138

IpaH1.4  AIGSGIMHQINNVYSLTPLSLPMELTPSCNEFYLKTWSEWEKNGTPGEQRN--IAFNRLKICLQNQEAE  75
IpaH9.8  ------MLPINNNFSLPQNSFYNTI-SGTYADYFSAWDKWEKQALPGEERD--EAVSRLKECLINNSDE  60
SspH1    EECTEFAEGASG--T---SLRPATTVSQKAAEYDAVWSKWERDAPAGESPGRAAVVQEMRDCLNNGNPV 199
SspH2    EEGTGHAEGASG--TFRTSFLPATTAPQTPAEYDAVWSAWRRAAPAEESRGRAAVVQKMRACLNNGNAV 205

IpaH1.4  LNLSELDLKTLPDL-PPQITTLEIRKNLLTHLPDLPPMLKVIHAQFNQL-------------------- 123
IpaH9.8  LRLDRLNLSSLPDNLPAQITL--------------------LNVSYNQL-------------------- 89
SspH1    LNVGASGLTTLPDRLPPHITTLVIPDNNLTSLPELPEGLRELEVSGNL-------------------QL 249
SspH2    LNVGESGLTTLPDCLPAHITTLVIPDNNLTSLPALPPELRTLEVSGNQLTSLPVLPPGLLELSIFSNPL 274

IpaH1.4  ESLPALPETLEELNAGDNKIKELPFLPENLTHLRVHNNRLHILPL--------------------LPPE 172
IpaH9.8  TNLPELPVTLKKLYSASNKLSELPVLPPALESLQVQHNELENLPA--------------------LPDS 138
SspH1    TSLPSLPQGLQKLWAYNNWLASLPTLPPGLGDLAVSNNQLTSLPE--------------------MPPA 298
SspH2    THLPALPSGLCKLWIFGNQLTSLPVLPPGLQELSVSDNQLASLPALPSELCKLWAYNNQLTSLPMLPSG 343

IpaH1.4  LKLLVVSGNRLDSIPPFPDKLEGLALANNFIEQLPEL------PFSMNR-------------------- 215
IpaH9.8  LLTMNISYNEIVSLPSLPQALKNLRATRNFLTELPAFSEGNN-PV-V-R-------------------- 184
SspH1    LRELRVSGNNLTSLPALPSGLQKLWAYNNRLTSLPEM------SPGLQELD------------------ 343
SspH2    LQELSVSDNQLASLPTLPSELYKLWAYNNRLTSLPAL------PSGLKELIVSGNRLTSLPVLPSELKE 406
                                              IpaH9.8 Amplifier loop

IpaH1.4  --------------------AVLMNNNLTTLPESVLRLAQNAFVNVAGNPLSGHTMRTLQQITTGPDYS 264
IpaH9.8  -------------------EYFFDRNQISHIPESILNLRNECSIHISDNPLSSHALPALQRLTSSPDYH 234
SspH1    ----------------------VSHNQLTRLPQSLTGLSSAARVYLDGNPLSVRTLQALRDII---GHS 387
SspH2    LMVSGNRLTSLPMLPSGLLSLSVYRNQLTRLPESLIHLSSETTVNLEGNPLSERTLQALREITSAPGYS 475

IpaH1.4  GPQIFFSMGNSATISAPEHSLADAVTAWFPENKQSD--VSQIWHAFEHEEHANTFSAFLDRLSDTVSAR 331
IpaH9.8  GPRIYFSMSDGQQN-TLHRPLADAVTAWFPENKQSD--VSQIWHAFEHEEHANTFSAFLDRLSDTVSAR 300
SspH1    GIRIHFDMAGPSVP-REARALHLAVADWLTSAREGEAAQADRWQAFGLEDNAAAFSLVLDRLRETENFK 455
SspH2    GPIIRFDMAGASAP-RETRALHLAAADWLVPAREGEPAPADRWHMFGQEDNADAFSLFLDRLSETENFI 543

IpaH1.4  NTSGFREQVAAWLEKLSASAELRQQSFAVAADATESCEDRVALTWNNLRKTLLVHQASEGLFDNDTGAL 400
IpaH9.8  NTSGFREQVAAWLEKLSASAELRQQSFAVAADATESCEDRVALTWNNLRKTLLVHQASEGLFDNDTGAL 369
SspH1    KDAGFKAQISSWLTQLAEDAALRAKTFAMATEATSTCEDRVTHALHQMNNVQLVHNAEKGEYDNNLQGL 524
SspH2    KDAGFKAQISSWLAQLAEDEALRANTFAMATEATSSCEDRVTFFLHQMKNVQLVHNAEKGQYDNDLAAL 612
                               catalytic cysteine | | catalytic acid

IpaH1.4  LSLGREMFRLEILEDIARDKVRTLHFVDEIEVYLAFQTMLAEKLQLSTAVKEMRFYGVSGVTANDLRTA 469
IpaH9.8  LSLGREMFRLEILEDIARDKVRTLHFVDEIEVYLAFQTMLAEKLQLSTAVKEMRFYGVSGVTANDLRTA 438
SspH1    VSTGREMFRLATLEQIAREKAGTLALVDDVEVYLAFQNKLKESLELTSVTSEMRFFDVSGVTVSDLQAA 593
SspH2    VATGREMFRLGKLEQIAREKVRTLALVDEIEVWLAYQNKLKKSLGLTSVTSEMRFFDVSGVTVTDLQDA 681
                                    |catalytic base

IpaH1.4  EAMVRSREENEFTDWFSLWGPWHAVLKRTEADRWAQAEEQKYEMLENEYSQRVADRLKASGLSGDADAE 538
IpaH9.8  EAMVRSREENEFTDWFSLWGPWHAVLKRTEADRWAQAEEQKYEMLENEYPQRVADRLKASGLSGDADAE 507
SspH1    ELQVKTAENSGFSKWILQWGPLHSVLERKVPERFNALREKQISDYEDTYRKLYDEVLKSSGLVDDTDAE 662
SspH2    ELQVKAAEKSEFREWILQWGPLHRVLERKAPERVNALREKQISDYEETYRMLSDTELRPSGLVGNTDAE 750
                                                    E2~Ub binding|

IpaH1.4  REAGAQVMRETEQQIYR---QLTDEVLALRLSENGSNHIA-                            575
IpaH9.8  REAGAQVMRETEQQIYR---QLTDEVLALRLPENGSQLHHS                            545
SspH1    RTIGVSAMDSAKKEFLDGLRALVDEVLGSYLTARWRLN---                            700
SspH2    RTIGARAMESAKKTFLDGLRPLVEEMLGSYLNVQWRRN---                            788
         |E2~Ub binding
```

B

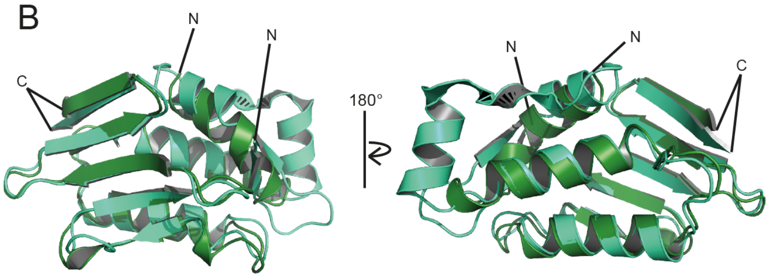

C

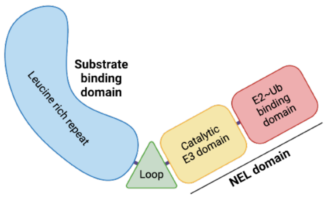

Figure S1 NEL protein sequence alignment.

A) Alignment of sequences of IpaH1.4, IpaH9.8, SspH1 and SspH2 using Clustal Omega, with SspH1 secondary structure shown above. N-terminal domains of SspH1 and SspH2 as predicted by AlphaFold2 are highlighted in green and turquoise respectively. SspH1 domains are highlighted in blue (LRR), green (flexible loop between LRR and NEL domains), yellow (E3 catalytic domain) and light red (E2-Ub binding domain). The catalytic cysteines, catalytic acids and bases are highlighted in red, and critical conserved residues highlighted in orange.[1] Grey residues represent those critical for E2-Ub binding.[1] B) Aligned AF2 predictions of N-terminal domains of SspH1 (green) and SspH2 (turquoise). C) Domain structure of NEL bacterial E3 ligases, with colours corresponding to domains highlighted in the sequence alignment.

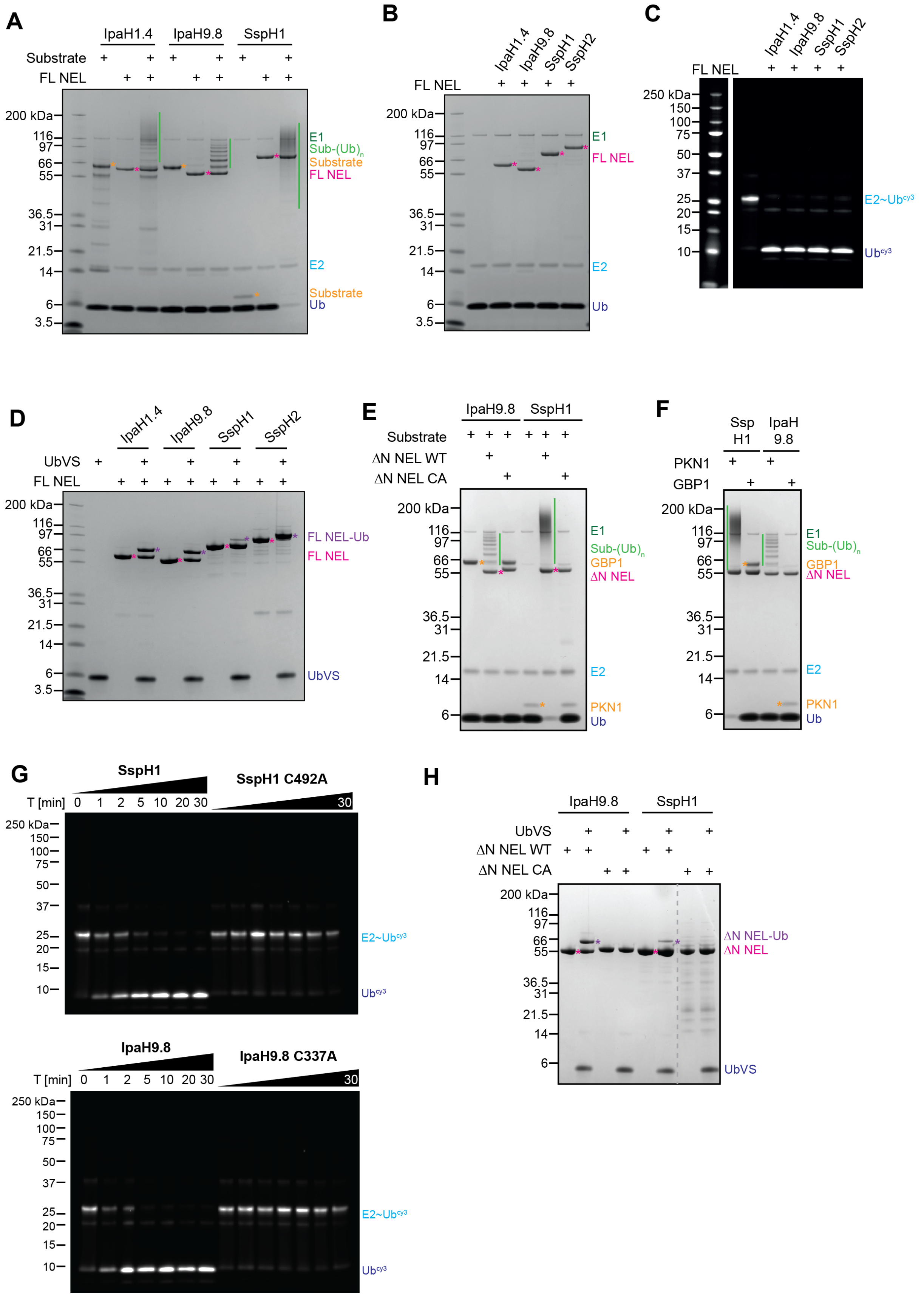

Figure S2 Full length (FL) and ΔNEL catalytic mutant assays.

A) Substrate ubiquitination assay with FL constructs of IpaH1.4 (substrate LUBAC), IpaH9.8 (substrate GBP1) and SspH1 (substrate HR1b PKN1). Performed with 0.1 μM UBA1 (E1), 2 μM UbcH5A (E2), 1.0 μM FL NEL (E3), 20 μM ubiquitin, 10 mM ATP at RT for 30 minutes with either 1 μM (LUBAC, GBP1) or 2 μM (HR1b PKN1) substrate.
B) Auto-ubiquitination assay with FL constructs of IpaH1.4, IpaH9.8, SspH1 and SspH2. Performed with 0.1 μM UBA1 (E1), 2 μM UbcH5A (E2), 1.0 μM FL NEL (E3), 20 μM ubiquitin, 10 mM ATP at RT for 30 minutes.
C) E2~Ub discharge assay with FL constructs of IpaH1.4, IpaH9.8, SspH1 and SspH2. Performed with 1 μM UbcH5A~Ub-cy3 (E2~Ub^{cy3}), 0.1 μM FL NEL (E3) at RT for 20 minutes.
D) Ubiquitin-loading assay with FL constructs of IpaH1.4, IpaH9.8, SspH1 and SspH2. Performed with 20 μM UbVS, 5 μM FL NEL (E3) at RT for 2 hours.
E) Substrate ubiquitination assay with ∆NEL constructs of IpaH9.8 and SspH1 either in the wild-type (WT) or with the catalytic mutant (CA; SspH1 C492A; IpaH9.8 C337A). Performed with 0.1 μM UBA1 (E1), 2 μM UbcH5A (E2), 1.0 μM FL NEL (E3), 20 μM ubiquitin, 10 mM ATP at RT for 30 minutes with either 1 μM (GBP1) or 2 μM (HR1b PKN1) substrate.
F) Substrate-swap ubiquitination assays performed as described in E).
G) E2~Ub discharge assay with constructs of ∆NEL constructs of IpaH9.8 and SspH1 either in the wild-type (WT) or with the catalytic mutant (SspH1 C492A; IpaH9.8 C337A). Performed with 1 μM UbcH5A~Ub-cy3 (E2~Ub^{cy3}), 5 nM IpaH9.8 or 50 nM SspH1 constructs at RT for 0-30 minutes.
H) Ubiquitin-loading assay with NEL domains of IpaH9.8 and SspH1 either in the wild-type (WT) or with the catalytic mutant (CA; SspH1 C492A; IpaH9.8 C337A). Performed with 20 μM UbVS, 5 μM NEL domain (E3) at RT for 2 hours.

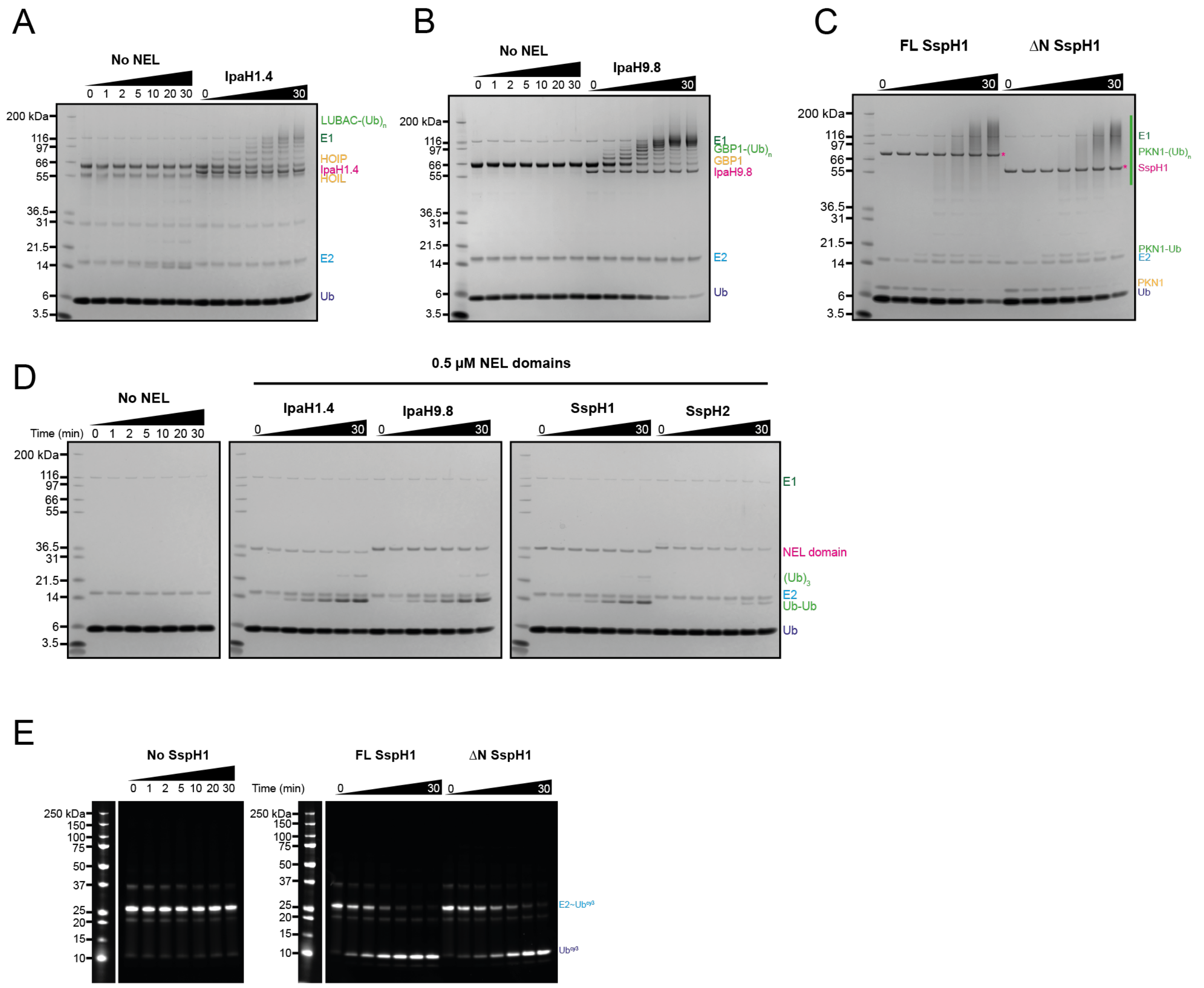

Figure S3 Time course assays.

A) Substrate ubiquitination assay with ΔN IpaH1.4 with LUBAC substrate. Performed with 0.1 µM UBA1 (E1), 2 µM UbcH5A (E2), 1 µM IpaH1.4 (E3), 1 µM LUBAC, 20 µM ubiquitin, 10 mM ATP at RT for 0-30 minutes.

B) Substrate ubiquitination assay with ΔN IpaH9.8 with GBP1 substrate. Performed with 0.1 µM UBA1 (E1), 2 µM UbcH5A (E2), 1.0 µM IpaH9.8 (E3), 1 µM GBP1, 20 µM ubiquitin, 10 mM ATP at RT for 0-30 minutes.

C) Substrate ubiquitination assay with FL SspH1 and ΔN SspH1 with HR1b PKN1 substrate. Performed with 0.1 µM UBA1 (E1), 2 µM UbcH5A (E2), 1.0 µM SspH1 (E3), 2 µM HR1b PKN1, 20 µM ubiquitin, 10 mM ATP at RT for 0-60 minutes.

D) Auto-ubiquitination assay with NEL domains of IpaH1.4, IpaH9.8, SspH1 and SspH2. Performed with 0.1 µM UBA1 (E1), 2 µM UbcH5A (E2), 0.5 µM NEL domain (E3), 20 µM ubiquitin, 10 mM ATP at RT for 0-30 minutes.

E) E2~Ub discharge assay with FL SspH1 and ΔN SspH1. Performed with 1 µM UbcH5A~Ub-cy3 (E2~Ub^{cy3}), 0.1 µM SspH1 (E3) at RT for 0-30 minutes.

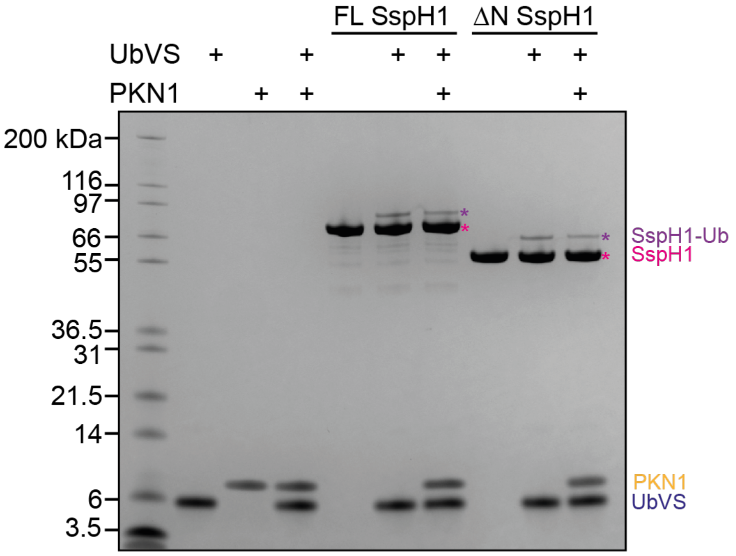

Figure S4 Loading of SspH1 ΔN and FL constructs with UbVS with and without PKN1.
A) Ubiquitin-loading assay with FL and ΔN SspH1, with and without HR1b PKN1. Performed with 20 µM UbVS, 5 µM FL NEL (E3), 10 µM HR1b PKN1 at RT for 2 hours.

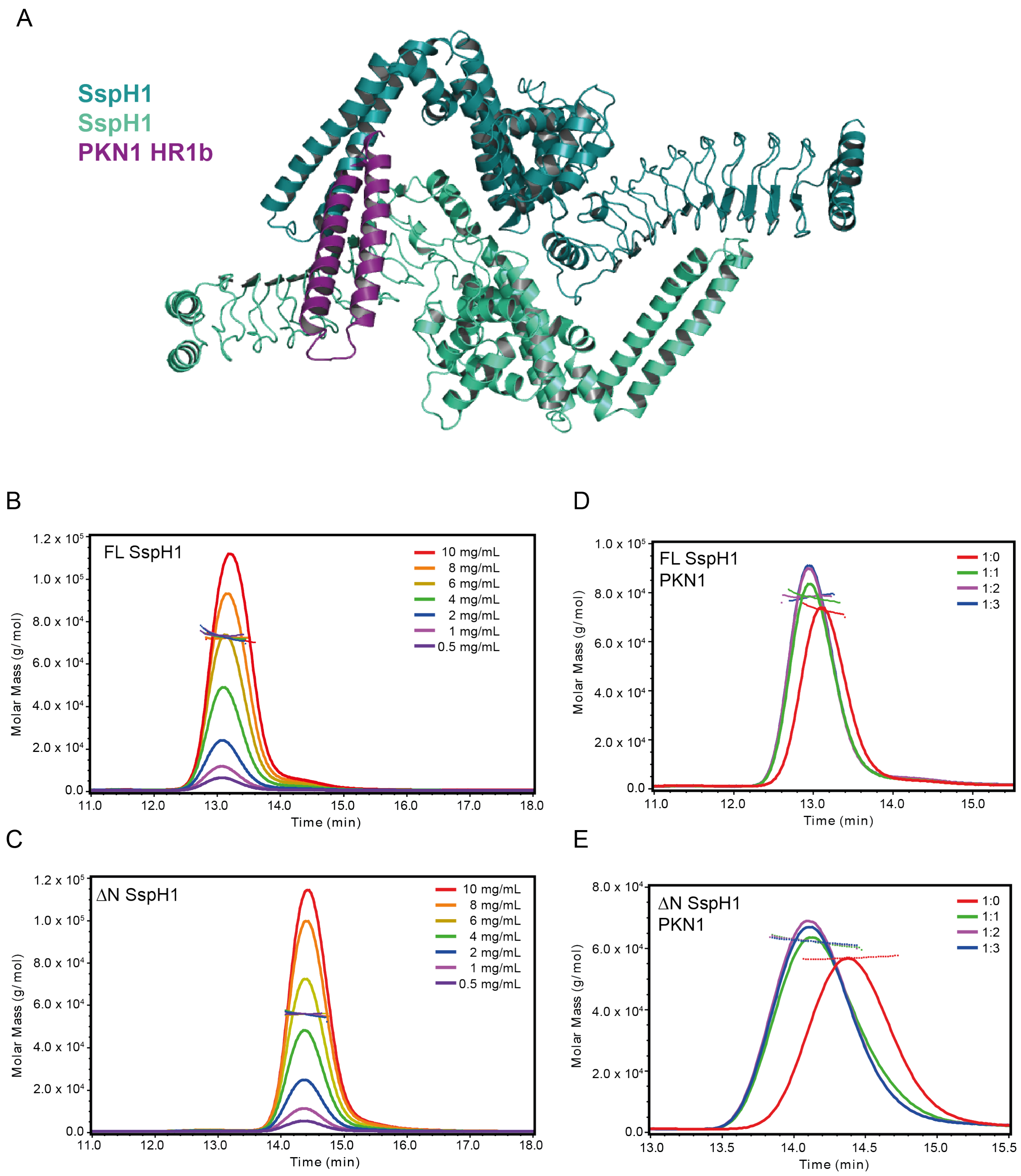

Figure S5 SspH1 is a monomer.

A) Symmetry related molecules of ΔNSspH1 (teal and turquoise) aligned to PKN1 HR1b (purple) in complex with SspH1 LRR domain (PDB 4NKG); SEC-MALLS of B) FL SspH1; C) ΔNSspH1; D) FL SSpH1 titrated with PKN1 HR1b; E) ΔNSspH1 titrated with PKN1 HR1b.

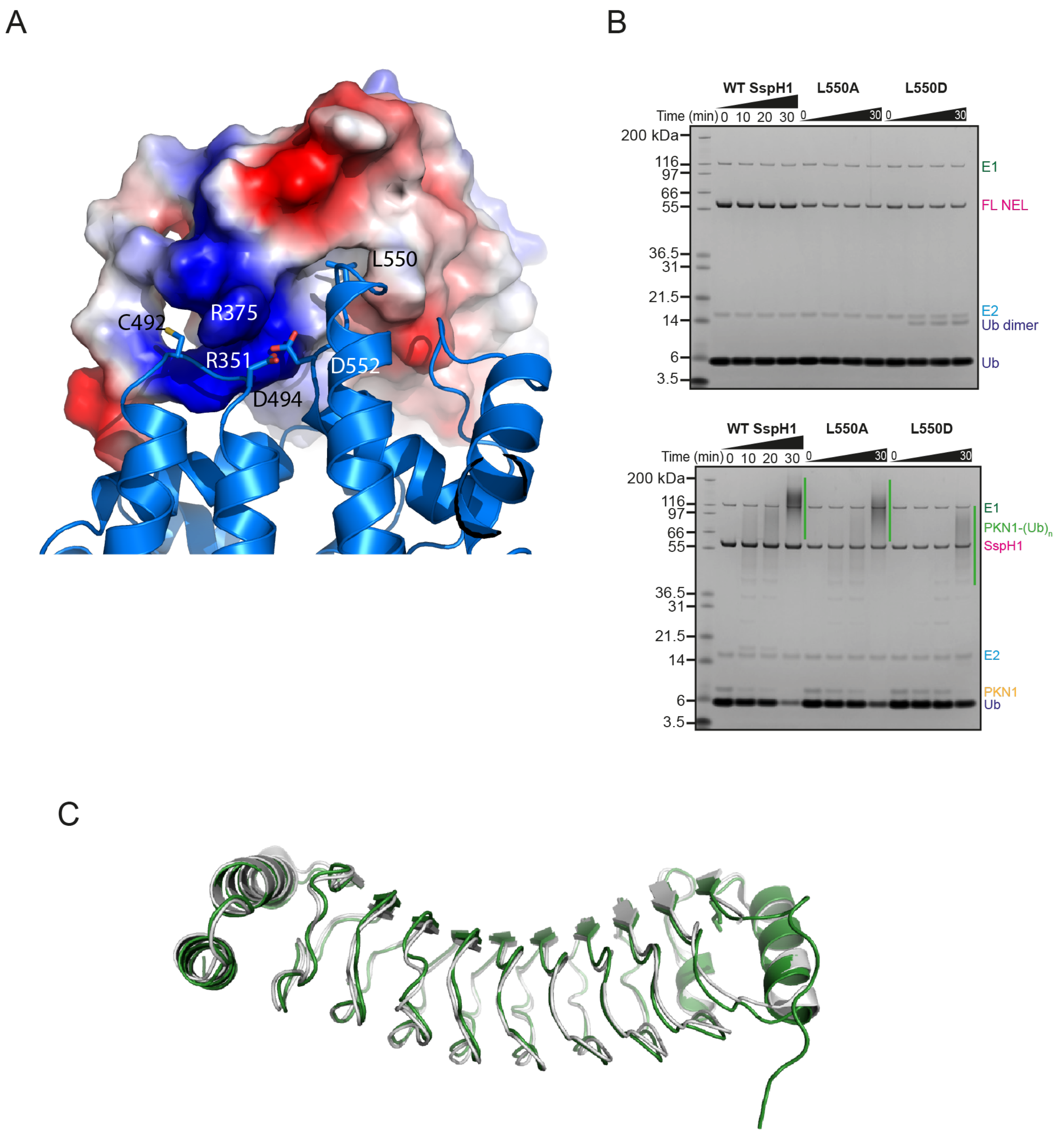

Figure S6 SspH1 mutant assays.

A) Close up view of the interface between LRR and NEL domain, with the NEL in surface representation coloured according to electrostatic potential and the NEL in cartoon representation. L550 points into a hydrophobic pocket formed by the LRR, whereas R351 and R375 from the LRR contact acidic residues of the NEL domain.

B) Substrate ubiquitination assay with ΔNSspH1 mutants L550A and L550D. Performed with 0.1 μM UBA1 (E1), 2 μM UbcH5A (E2), 1.0 μM SspH1 (E3), 2 μM HR1b PKN1, 20 μM ubiquitin, 10 mM ATP at RT for 0-30 minutes.

C) Overlap of the LRR domains of the SspH1 structure presented here in green and the LRR domain structure bound to PKN1 HR1b (PDB: 4NKG) in grey[2].

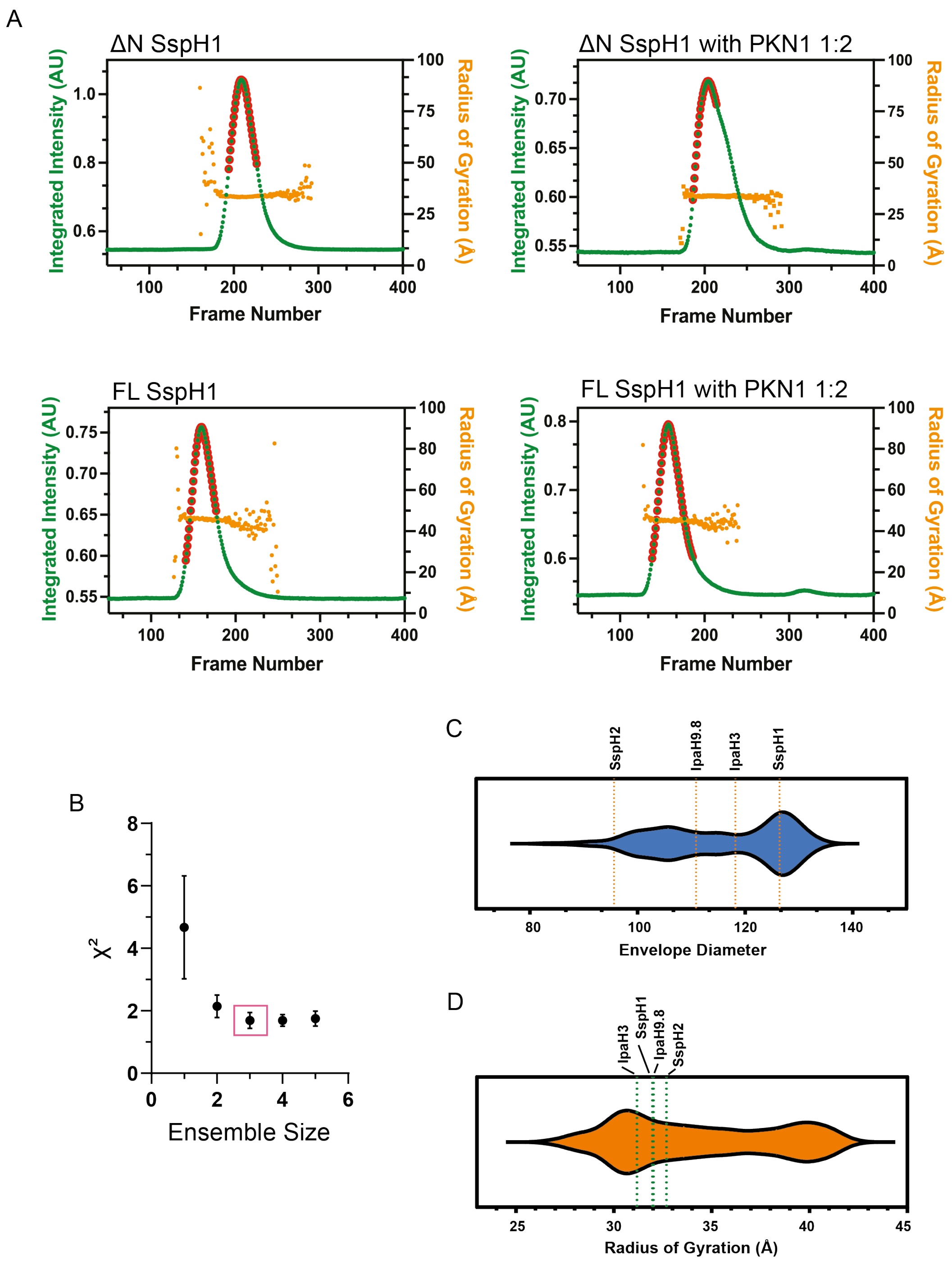

Figure S7 SEC-SAXS analysis and Xplor-NIH modelling.

A) Integrated intensities of recorded SAXS as a function of frames recorded off the size-exclusion column. The orange points are the values of the radius of gyration of the eluting species along the gel-filtration peak. Highlighted in red are the averaged frames for the final scatting curve. B) SAXS-based Xplor-NIH calculated ensemble χ^2 values versus the size of the ensemble. The lowest χ^2 values are achieved with a minimum ensemble size made of 3 conformers. C) Envelope diameter and radius of gyration distributions of the calculated ensemble conformers with χ^2 values smaller than 1.5. The values for the available full-length structures of SspH2 (PDB 3G06), IpaH9.8 (PDB 6LOL), IpaH3 (PDB 3CVR) are reported with the values obtained for our crystal structure.

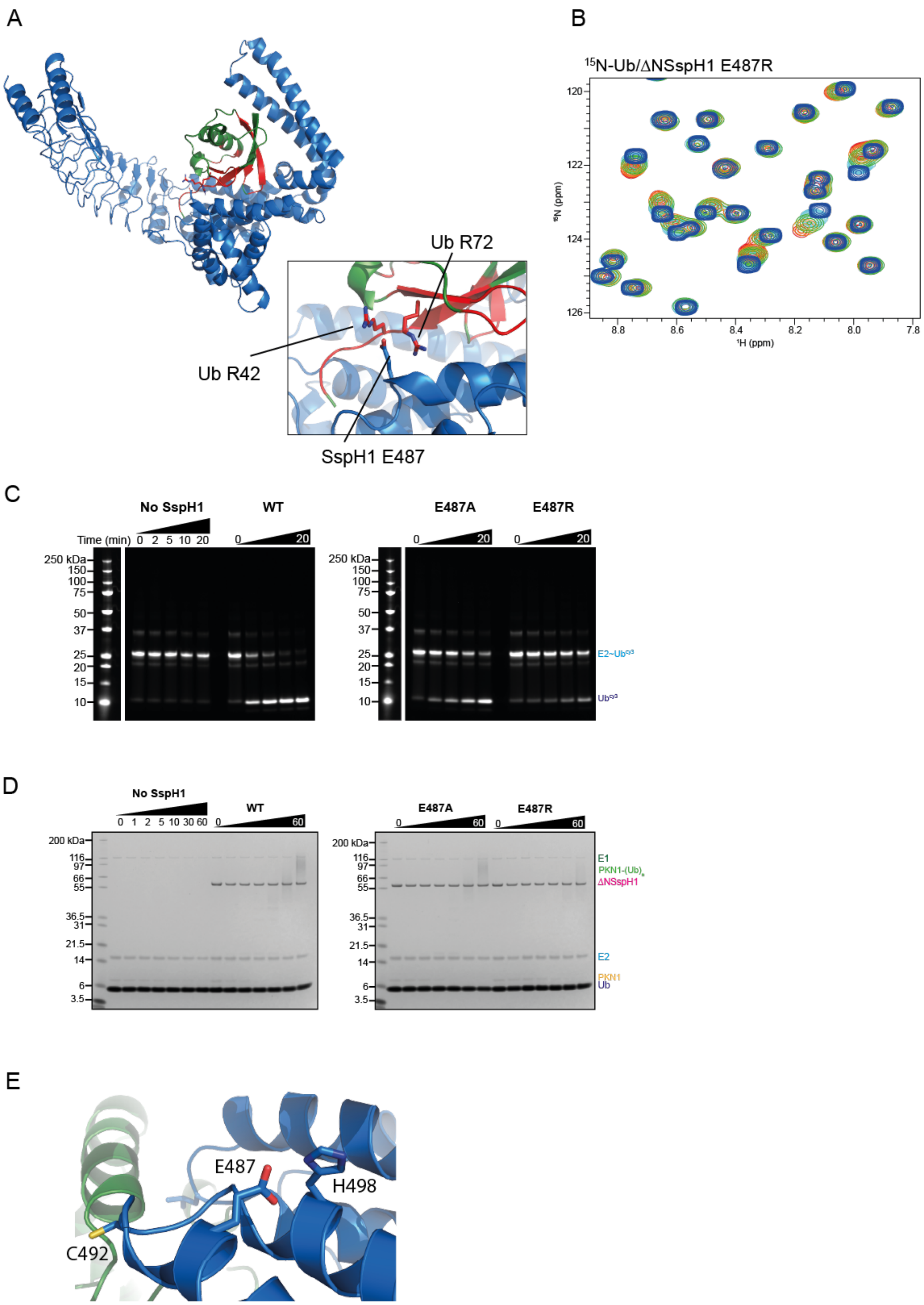
A
B
C
D
E
Ub R72
Ub R42
SspH1 E487
15N-Ub/ΔNSspH1 E487R
120
122
124
126
15N (ppm)
8.8
8.6
8.4
8.2
8.0
7.8
1H (ppm)
No SspH1
WT
E487A
E487R
Time (min)
0 2 5 10 20
0
20
250 kDa
150
100
75
50
37
25
20
15
10
0 1 2 5 10 30 60
60
200 kDa
116
97
66
55
36.5
31
21.5
14
6
3.5
E1
PKN1-(Ub)n
ΔNSspH1
E2
PKN1
Ub
E487
H498
C492

Figure S8 Analysis of the SspH1-ubiquitin interaction.

A) Cartoon representation of the top-ranking AF3 model complex of ubiquitin (green) bound to ∆NSspH1 (blue). Residues experiencing chemical shift perturbation in the NMR titration of ^{15}N-labelled ubiquitin with ΔNSspH1 are shown in red on the ubiquitin molecule. Insert: SspH1 E487 is predicted to form interactions with perturbed residues R72 and R42 of ubiquitin.

B) Details of the titration of ^{15}N-labeled ubiquitin titrated with ∆NSspH1 E487R. Spectra at different ligand concentrations are plotted at the same contour level.

C) E2~Ub discharge assay with ∆NSspH1 E487A and E487R mutants. Performed with 1 µM UbcH5A~Ub-cy3 (E2~Ub^{cy3}), 0.1 µM SspH1 (E3) at RT for 0-30 minutes.

D) Substrate ubiquitination assay with ∆NSspH1 E487A and E487R mutants with HR1b PKN1 substrate. Performed with 0.1 µM UBA1 (E1), 2 µM UbcH5A (E2), 1.0 µM SspH1 (E3), 2 µM HR1b PKN1, 20 µM ubiquitin, 10 mM ATP at RT for 0-30 minutes.

E) Close up of the interaction between E487 and H498 which are positioned in the helices either side of the loop containing the catalytic cysteine.

Table S1. Data collection and refinement statistics.

	SspH1 161-700
Wavelength (Å)	0.62
Resolution range	58.31 - 2.9 (3.0 - 2.9)
Space group	P 6 2 2
Unit cell	170.8 170.8 94.8 90 90 120
Total reflections	735501 (73343)
Unique reflections	18583 (1811)
Multiplicity	39.6 (40.5)
Completeness (%)	99.8 (99.6)
Mean I/sigma(I)	6.8 (0.4)
Wilson B-factor	74.61
R-merge	0.4992 (6.506)
R-meas	0.5056 (6.588)
R-pim	0.07988 (1.03)
CC1/2	0.996 (0.416)
CC*	0.999 (0.767)
Reflections used in refinement	18544 (1804)
Reflections used for R-free	900 (105)
R-work	0.2595 (0.3720)
R-free	0.3158 (0.4481)
CC(work)	0.943 (0.630)
CC(free)	0.832 (0.497)
Number of non-hydrogen atoms	4247
macromolecules	4193
ligands	14
solvent	44
Protein residues	540
RMS(bonds)	0.001
RMS(angles)	0.36
Ramachandran favored (%)	98.88
Ramachandran allowed (%)	1.12
Ramachandran outliers (%)	0.00
Rotamer outliers (%)	0.66
Clashscore	18.89
Average B-factor	78.93
macromolecules	79.16
ligands	59.55
solvent	61.58

Statistics for the highest-resolution shell are shown in parentheses.

Table S2: SAXS parameters and data analysis software.

Supplementary Table 1: SAXS parameters.

**Data collection**				
Beamline	SWING at Soleil			
q range (Å^{-1})	0.0082–0.66			
Detector	EigerX4M in vacuum			
Column	Bio-SEC 3 Agilent 300 Å			
Flow rate (ml/min)	0.3			
Temperature (°C)	15			
**Samples details**	**ΔNSspH1**	**ΔNSspH1/PKN1**	**SspH1**	**SspH1/PKN1**
Sample volume (µl)	60	60	60	60
Sample concentration (mg/ml)	10.0	11.5	8.0	9.0
**Structural parameters**				
**Reciprocal Space**				
Rg (Å) Guinier	34.5	35.1	48.2	47.7
I(0) (cm^{-1})	0.11	0.09057	0.01101	0.02078
qRg limit	1.05	1.04	1.16	0.97
**Real Space**				
Rg (Å) P(R)	34.6 ± 0.01	35.2 ± 0.05	48.5 ± 0.1	47.8 ± 0.08
I(0) (cm^{-1})	0.11000 ± 0.00003	0.09058 ± 0.00009	0.01102 ± 0.00001	0.02078 ± 0.00001
Rc (Å)	19.6	20.7	18.6	20.2
Dmax (Å)	114	124	189	198
Porod volume (Å^3)	86317	104099	113536	143322
**Molecular mass determination**				
Theoretical MW (kDa)	59.5	68.8	77.1	86.4
DATPOROD MW (kDa) (Vp/1.6)	53.9	65.1	71.0	89.6
SAXS MoW2 (q = 0.3 Å^{-1})	61.9	70.1	82.9	91.9

**Data analysis software**	
Primary Data Reduction	Foxtrot
Data processing	Primus & Scatter
Computation of scattering intensities	Crysol
Structural modelling	Xplor-NIH
3D graphics representation	Pymol

References

1. Keszei, A. F. A. & Sicheri, F. Mechanism of catalysis, E2 recognition & autoinhibition for the IpaH family of bacterial E3 ubiquitin ligases. *Proc Natl Acad Sci U S A* 114, 1311–1316 (2017).
2. Keszei, A. F. A. *et al.* Structure of an SspH1-PKN1 Complex Reveals the Basis for Host Substrate Recognition and Mechanism of Activation for a Bacterial E3 Ubiquitin Ligase. *Mol Cell Biol* 34, 362–373 (2014).
